# A Rare Occurrence of Uterine Perforation Following the Dilation and Curettage for Missed Abortion

**DOI:** 10.7759/cureus.70079

**Published:** 2024-09-24

**Authors:** Sakshi S Dudhe, Shubham Waghulkar, Gaurav V Mishra, Pratapsingh Parihar, Devyansh Nimodia

**Affiliations:** 1 Radiodiagnosis, Jawaharlal Nehru Medical College, Datta Meghe Institute of Higher Education and Research, Wardha, IND

**Keywords:** abortion, d&c, dilatation and curettage, hemoperitoneum, uterine perforation

## Abstract

Uterine perforation represents an uncommon yet potentially significant complication that may arise during the process of dilation and curettage (D&C), a frequently conducted procedure employed for a variety of gynaecological indications, including missed abortion. This report delineates an atypical case of uterine perforation in a 30-year-old female patient who underwent D&C subsequent to a missed abortion at 10 weeks of gestation and subsequently experienced acute abdominal discomfort accompanied by clinical features of internal haemorrhage shortly after the intervention. Diagnostic imaging corroborated the occurrence of uterine perforation, prompting the execution of emergency surgery to rectify the perforation and address concomitant complications. Following the surgical intervention, the patient achieved a complete recovery.

This case report underscores the necessity for heightened vigilance during D&C procedures. The early identification and swift surgical management of this condition are imperative to avert severe morbidity or mortality associated with this infrequent complication. Furthermore, it accentuates the importance of pre-operative counselling and post-operative surveillance to ensure the timely identification and management of potential complications.

## Introduction

Uterine curettage has been a universally accepted procedure since 1843 [1]. For the management of events like retained products of conception, post-abortions, and termination of pregnancy, dilation and curettage (D&C) is a procedure performed. It is considered to be comparatively safer. D&C has proven to have lesser rates of perforation of the uterus, approximately 0.05% in the first trimester and 0.32% in the second trimester of pregnancy [2]. Though highly invasive, D&C is one of the most commonly performed procedures in gynaecology. The risk of uterine perforation is increased depending upon parity, advanced age, and general anaesthesia. Perforation can often be misdiagnosed due to low expectations of this particular complication. Bladder and rectal injuries occur in almost 15% of these cases [3].

The uterine rupture has a dramatic presentation of continuous abdominal pain, vaginal bleeding, extrusion of the product of pregnancy through the uterine rupture, and bleeding into the abdominal cavity [4]. Rupture in primigravida is rare [5]. An incidence of uterine rupture is 0.006-0.012% in a healthy, unscarred uterus [6]. Perforations at the internal os and lower uterus are more dangerous as they can involve uterine vessels [7]. Mechanical cervical dilation or the insertion of a sharp uterine instrument causes uterine perforation. Risk factors of uterine perforation include uterine anomalies, maternal infections, recent pregnancy, and post-menopausal status. Uterine rupture causing hemoperitoneum in an unscarred uterus is a rare occurrence. Depending on the perforation site and size, patients can present with pain, vaginal bleeding, hematuria, hypotension, and weakness. Perforations are generally small, involving the anterior wall of the uterus and uterine fundus. Rarely, the internal cervical os and the lower uterine segment, if perforated, involve the surrounding parauterine vessels, causing potentially dangerous outcomes [8]. Hemoperitoneum caused by uterine perforation is a very rare and serious complication following D&C, particularly in cases of spontaneous or therapeutic abortion. Uterine perforation can lead to significant morbidity and requires prompt diagnosis and management to prevent serious consequences. We herein report a case of a 30-year-old woman with uterine rupture after D&C, which caused hemoperitoneum.

## Case presentation

A 30-year-old gravida 2, para 2 female presented to the emergency department with severe acute abdominal pain following a D&C performed three hours earlier at an outside hospital. She also reported moderate vaginal bleeding post-procedure. The patient had a history of missed abortion at 10 weeks of gestation, for which the D&C was performed. Her obstetric history included two full-term normal vaginal deliveries, with no history of cesarean section or uterine surgery. The patient was stabilized with intravenous fluids, analgesics, and antibiotics.

The D&C procedure was uneventful; however, approximately three hours post-procedure, the patient began experiencing severe lower abdominal pain, dizziness, and nausea. She appeared pale and diaphoretic. Her blood pressure dropped to 90/60 mmHg, with a heart rate increase of 126 beats per minute, a respiratory rate of 22 breaths per minute, and a body temperature of 36.5°C. The patient had no history of allergies. A systemic examination revealed a distended abdomen with severe tenderness and rebound tenderness in the lower abdomen. Bowel sounds were sluggish, and dullness over the flanks suggested possible fluid accumulation. Pelvic examination showed a closed cervical os with no active bleeding, but tenderness was noted on bimanual examination. Emergency investigations included a complete blood count (CBC), which showed a haemoglobin level of 8 g/dL (down from a pre-operative level of 12 g/dL), a white blood cell count of 29,100/mm^3^, and a platelet count of 294,000/mm^3^.

**Figure 1:**
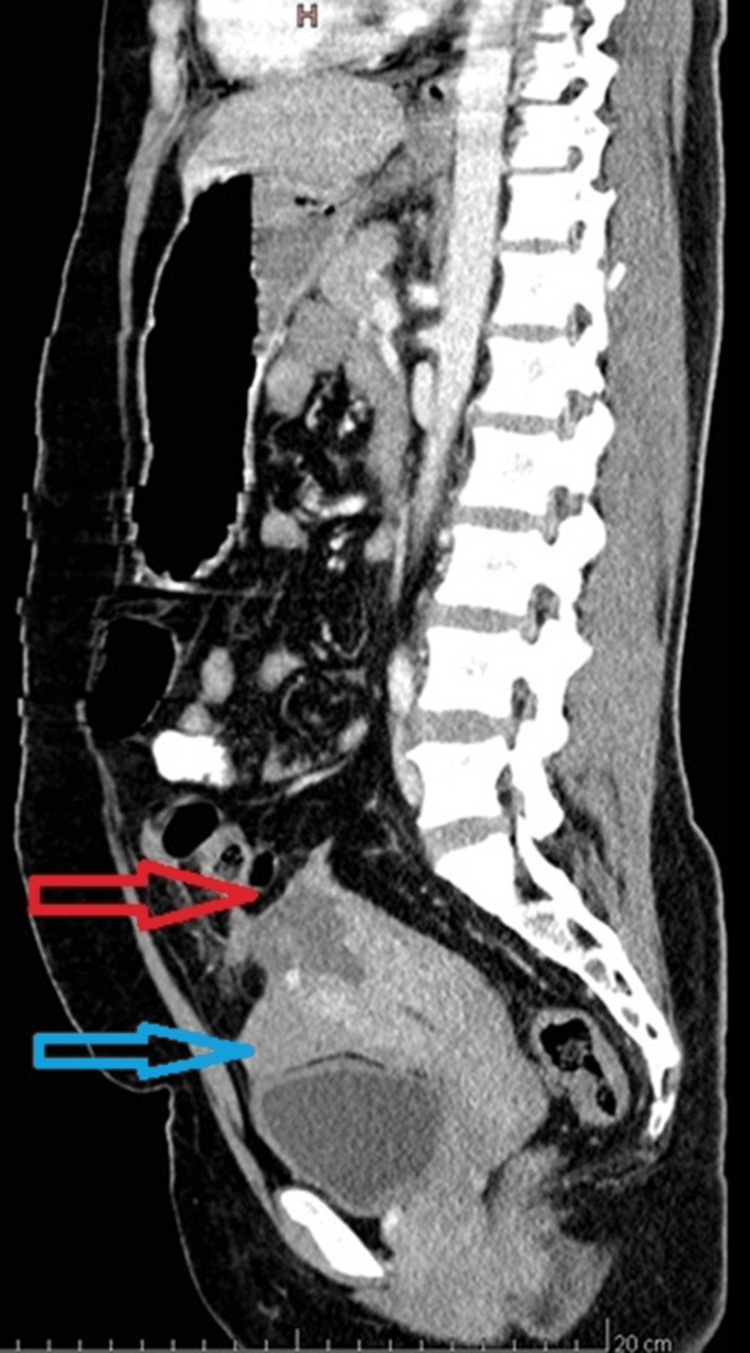
Contrast-enhanced CT (CECT) scan of the abdomen in the sagittal section (arterial phase) showing a 3.8 x 3.2 cm defect at the uterine fundus (red arrow). Free fluid with a CT attenuation value of +38 to +55 Hounsfield units (HU), suggestive of hemoperitoneum, is also visible (blue arrow).

**Figure 2:**
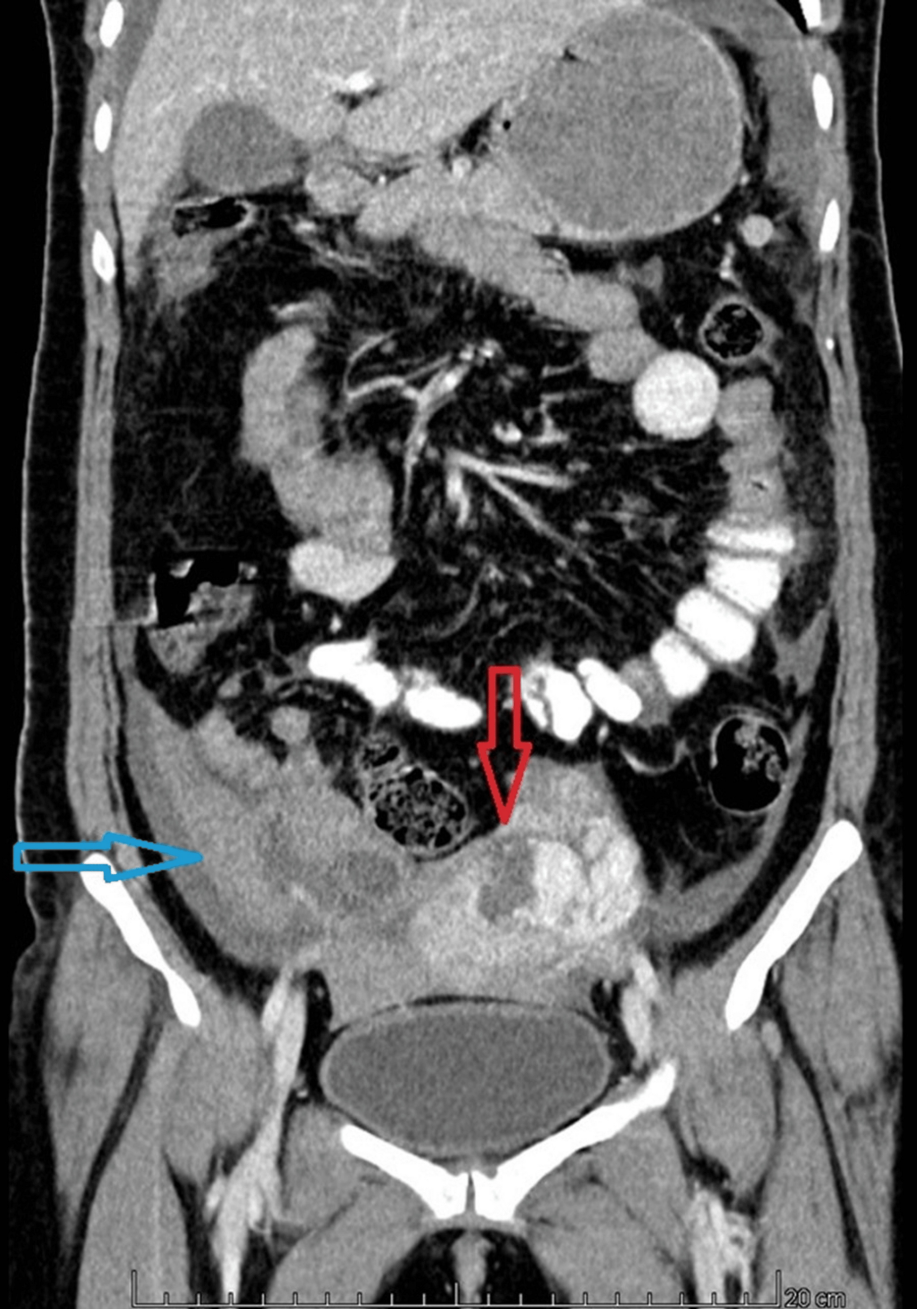
Contrast-enhanced CT (CECT) scan of the abdomen in the coronal section showing a 3.8 x 3.2 cm defect at the uterine fundus (red arrow), suggestive of uterine perforation. Free fluid with a CT attenuation value of +38 to +55 Hounsfield units (HU) is noted on the right side of the pelvis, indicative of hemoperitoneum (blue arrow).

**Figure 3:**
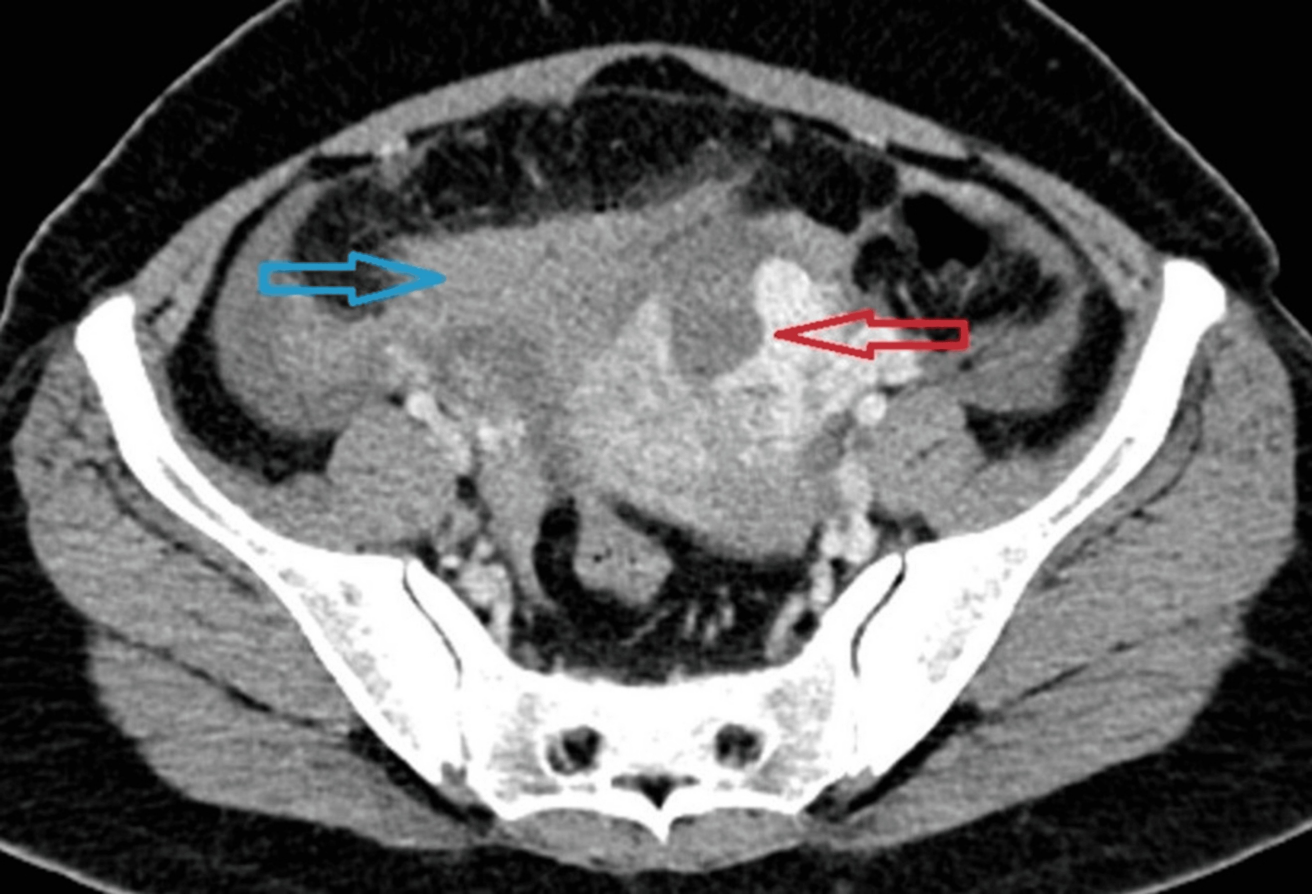
Contrast-enhanced CT (CECT) scan of the abdomen in the axial section (arterial phase) showing a uterine rupture at the fundus (red arrow) and evidence of hemoperitoneum (blue arrow).

An emergency ultrasound (US) was done which showed free fluid in the abdominal cavity with a CT attenuation value of +38 to +55 Hounsfield units (HU), suggestive of hemoperitoneum. An irregular uterine contour with a defect of size 3.8 x 3.2cm was noted at the fundus of the uterus, favouring perforation. A provisional diagnosis of uterine perforation with internal bleeding was made. No retained products of conception were noted. Contrast-enhanced computed tomography (CECT) was advised which revealed a defect in uterine myometrium at the fundus part and high attenuation-free fluid in the pelvis, confirming the finding of uterine rupture and hemoperitoneum, as shown in Figures 1-3.

An exploratory laparotomy was done, and the abdominal wall was opened by vertical incision and showed a moderate amount of fresh blood. A total of 1600 cc of mixed-density fluid was suctioned. The uterus was detected to be ruptured in the fundus part and was repaired in three layers with the help of absorbable sutures. Hemostasis was achieved, and the abdominal cavity was thoroughly irrigated. The total blood loss was estimated to be more than two litres. No complications arose during the post-operative hospital stay, and the patient was closely monitored. She was given blood transfusions. The patient's condition stabilized over the next 48 hours. Her haemoglobin levels gradually improved, and her vital signs returned to normal. She received broad-spectrum antibiotics for five days and was gradually transitioned to oral intake. She was discharged after five days with instructions for follow-up.

## Discussion

Uterine perforation is a fatal obstetric emergency. Per 10,000 deliveries, the incidence of uterine rupture is found to be 5.3, according to the World Health Organization (WHO). Uterine rupture is associated with high materno-fetal mortality and morbidity. D&C is a common gynaecological procedure, that is performed for various diagnostic purposes and to manage incomplete abortions, missed abortions, or therapeutic abortions. This procedure has its own risks, like infection and bleeding. Uterine perforation and hemoperitoneum are rare post-D&C complications. Hemoperitoneum resulting from uterine rupture in an unscarred uterus is a rare occurrence and a potentially fatal situation. Maternal mortality in uterine rupture ranges from 1% to 13% [2].

Advanced gestational age, multiparity, and surgical inexperience are risk factors for uterine perforation [2]. The most common risk factor for rupture of the uterus is previous caesarean [9]. A low incidence of uterine perforation has been noted, which is approximately 0.8-6.4 per 1000 procedures [3]. Uterine rupture is comparatively more common in developing countries. Risk factors include previous uterine surgery, including caesarean section, laparotomy, laparoscopic surgery, hysteroscopic surgery, iatrogenically caused uterine perforation, multiparity, previous history of instrumental abortion, improper labour augmentation, fundal pressure, placenta accreta, traumatic causes, and congenital anomalies of uterine. According to aetiology, uterine rupture is classified as (1) spontaneous rupture of a previous scar due to a cesarean section or myomectomy; (2) traumatic rupture of a previous scar due to obstetric or accidental causes; (3) spontaneous rupture of an unscarred uterus; and (4) traumatic rupture of an unscarred uterus [10].

Whenever the operating instrument passes beyond the expected length of the uterus, perforation should be suspected. Uterine perforation with other internal organ injuries can be suspected if there is too much bleeding, hematuria and fat or omentum in the suction cannula. The Intraabdominal or retroperitoneal haemorrhage causes perioperative hypotension as its first sign [11]. Post-procedural uterine perforations are generally observed in six months [12]. In recent years, increases in the rate of caesarean delivery (CS), prostaglandin usage and vaginal delivery following caesareans have led to cases of uterine rupture [13]. Uterine rupture risk in a previous lower segment caesarean procedure is approximately 0.2-1.5% [9].

A serious but extremely rare complication of D&C is uterine perforation. The uterine fundus is the most commonly perforated area. During procedures such as D&C, perforations are often undetected or unrecognized, leading to serious complications. Intestinal injuries can occur for which surgical intervention is needed. The small intestine, appendix or omentum can enter the uterine cavity due to uterine perforation [14].

Considerable morbidity and mortality may arise from uterine perforation and rupture, whether due to iatrogenic or non-iatrogenic causes of uterine wall trauma. Radiological imaging is essential in diagnosing as well as in the identification of the exact site of perforation. US is the primary imaging modality of choice, particularly for detecting uterine wall damage. To confirm US findings and other complications like bowel injury, CT and magnetic resonance imaging (MRI) can be used as adjuncts if needed. Moreover, it provides insight into the precise location of the perforation [6].

Treatment of uterine rupture includes prompt surgical intervention and hemodynamic stabilization. Correction of hypovolemia, proper airway and oxygen supply are necessary. Maternal mortality is 0.44%. Potential complications encompass haemorrhage, shock, infection, intravascular coagulation, paralytic ileus, pulmonary emboli, kidney failure and peritonitis. The technique employed and the level of operative expertise also impact the outcomes of D&C [10]. Technique and operative experience also affect the outcome of D&C [3]. Poor outcomes can be due to delays in patient diagnosis, poor transportation facilities and other causes like low socioeconomic status, ignorance and low patient haemoglobin. Differential diagnoses of uterine rupture include placental abruption, placenta previa, uterine inversion, cervical or vaginal tear, coagulopathy, atonic uterus, and uterine artery rupture [6].

Uterine perforation is an infrequent yet significant adverse event associated with D&C, especially in cases of missed abortion. The present instance underscores the significance of timely detection and intervention. The manifestation of hemoperitoneum secondary to uterine perforation may include sudden onset of abdominal pain and low blood pressure. Timely surgical intervention plays a critical role in achieving positive prognostic results.

## Conclusions

This case emphasizes the importance of increased awareness regarding potential complications following D&C, as well as the significance of prompt surgical intervention in the treatment of uterine perforation accompanied by hemoperitoneum. Although uncommon, uterine perforation and rupture may arise as significant complications subsequent to procedures. Various imaging techniques can identify the location, extent, and associated complications of uterine perforation and rupture. It is imperative for radiologists to possess a comprehensive understanding and awareness of these imaging findings to ensure timely and accurate diagnosis, enabling the monitoring of potential further complications. The radiologist assumes a crucial role in supporting the clinical team by supplying diagnostic insights and supplementary information essential for effective treatment planning and care. Consistent post-procedural follow-ups and vigilant monitoring can facilitate early recognition and management of such life-threatening complications.

## References

[1] Melvin RH, Korman W (1963). Uterine perforation during dilatation and curettage. Obstet Gynecol.

[2] Aboughalia H, Basavalingu D, Revzin MV, Sienas LE, Katz DS, Moshiri M (2021). Imaging evaluation of uterine perforation and rupture. Abdom Radiol (NY).

[3] Zorilă GL, Căpitănescu RG, Drăgușin RC (2023). Uterine perforation as a complication of the intrauterine procedures causing omentum incarceration: a review. Diagnostics (Basel).

[4] Ghahramani L, Moslemi S, Tahamtan M, Hashemizadeh MH, Keshavarzi A (2013). Antepartum uterine rupture occurring at the site of a previously repaired dilatation and curettage-induced perforation: a case report. Bull Emerg Trauma.

[5] Padhye SM (2007). Rupture uterus in primigravida: morbidity and mortality. Kathmandu Univ Med J (KUMJ).

[6] Tayade S, Chadha A, Khandelwal S, Makhija N, Tilva H, Madaan S (2022). Uterine rupture following non-operative vaginal delivery: a close save of delayed presentation with hemoperitoneum to a rural tertiary care hospital. Cureus.

[7] Shakir F, Diab Y (2013). The perforated uterus. Obstet Gynaecol.

[8] Levy BS, Falcone T, Chakrabarti A (2023). Uterine perforation during gynecological procedures. UpToDate.

[9] Deka D, Bahadur A, Dadhwal V, Gurunath S, Vaid A (2011). Successful outcome in pregnancy complicated by prior uterine rupture: a report of two cases. Arch Gynecol Obstet.

[10] Pontis A, Prasciolu C, Litta P, Angioni S (2016). Uterine rupture in pregnancy: two case reports and review of literature. Clin Exp Obstet Gynecol.

[11] Pillai KS, Bhuvana S, Vijayaraghavan J, Alexander N (2014). A case of uterine perforation with successful conservative laparoscopic management. IJRTST.

[12] Chen LH, Lai SF, Lee WH, Leong NK (1995). Uterine perforation during elective first trimester abortions: a 13-year review. Singapore Med J.

[13] Usta IM, Hamdi MA, Musa AA, Nassar AH (2007). Pregnancy outcome in patients with previous uterine rupture. Acta Obstet Gynecol Scand.

[14] Su S, Tao G, Dong B, Shi L, Dong J (2015). Delayed presentation of uterine perforation with ovary migration after dilatation and curettage. Int J Clin Exp Med.

